# Combinatorial Strategies for the Induction of Immunogenic Cell Death

**DOI:** 10.3389/fimmu.2015.00187

**Published:** 2015-04-24

**Authors:** Lucillia Bezu, Ligia C. Gomes-da-Silva, Heleen Dewitte, Karine Breckpot, Jitka Fucikova, Radek Spisek, Lorenzo Galluzzi, Oliver Kepp, Guido Kroemer

**Affiliations:** ^1^Equipe 11 labellisée par la Ligue Nationale contre le Cancer, Centre de Recherche des Cordeliers, Paris, France; ^2^U1138, INSERM, Paris, France; ^3^Metabolomics and Cell Biology Platforms, Gustave Roussy Campus Cancer, Villejuif, France; ^4^Faculté de Medecine, Université Paris-Sud, Le Kremlin-Bicêtre, France; ^5^Department of Chemistry, University of Coimbra, Coimbra, Portugal; ^6^Laboratory for General Biochemistry and Physical Pharmacy, Faculty of Pharmacy, Ghent University, Ghent, Belgium; ^7^Laboratory of Molecular and Cellular Therapy, Vrije Universiteit Brussel, Jette, Belgium; ^8^Sotio a.c., Prague, Czech Republic; ^9^Department of Immunology, 2nd Faculty of Medicine, University Hospital Motol, Charles University, Prague, Czech Republic; ^10^Gustave Roussy Campus Cancer, Villejuif, France; ^11^Université Paris Descartes, Paris, France; ^12^Université Pierre et Marie Curie, Paris, France; ^13^Pôle de Biologie, Hopitâl Européen George Pompidou, AP-HP, Paris, France

**Keywords:** ATP, autophagy, calreticulin, endoplasmic reticulum stress, HMGB1, type I interferon

## Abstract

The term “immunogenic cell death” (ICD) is commonly employed to indicate a peculiar instance of regulated cell death (RCD) that engages the adaptive arm of the immune system. The inoculation of cancer cells undergoing ICD into immunocompetent animals elicits a specific immune response associated with the establishment of immunological memory. Only a few agents are intrinsically endowed with the ability to trigger ICD. These include a few chemotherapeutics that are routinely employed in the clinic, like doxorubicin, mitoxantrone, oxaliplatin, and cyclophosphamide, as well as some agents that have not yet been approved for use in humans. Accumulating clinical data indicate that the activation of adaptive immune responses against dying cancer cells is associated with improved disease outcome in patients affected by various neoplasms. Thus, novel therapeutic regimens that trigger ICD are urgently awaited. Here, we discuss current combinatorial approaches to convert otherwise non-immunogenic instances of RCD into *bona fide* ICD.

## Introduction

The expression “immunogenic cell death” (ICD) generally refers to a functionally peculiar case of regulated cell death (RCD) that – in immunocompetent hosts – is capable of activating an adaptive immune response against dead cell-associated antigens (1–5). Of note, ICD generally (but not obligatorily) manifests with apoptotic morphological features, and at least some of its manifestations depend on components of the apoptotic apparatus (6–8). Irrespective of these morphological and biochemical considerations, immunocompetent mice injected s.c. with cancer cells succumbing to *bona fide* ICD (in the absence of any adjuvant) develop a cellular immune response associated with the establishment of immunological memory that protects them from a subsequent challenge with living cells of the same type (1–3). Importantly, vaccination experiments of this type, involving murine cells and syngeneic mice, remain the gold-standard method to identify *bona fide* ICD, though several tests have been developed to detect some of its cellular manifestations (see below) (2, 3, 9, 10).

Only a few lethal stimuli are intrinsically endowed with the ability to trigger ICD (9, 11–14). These include some chemotherapeutic agents that are employed in the clinic, including (1) various anthracyclines (i.e., doxorubicin, epirubicin, and idarubicin), which are commonly used against a wide panel of malignant conditions (15–17); (2) mitoxantrone, an anthracenedione generally used for the treatment of acute myeloid leukemia, breast carcinoma, non-Hodgkin’s lymphoma, and prostate carcinoma (15, 16); (3) oxaliplatin, a platinum derivative approved for use in combination with 5-fluorouracil to treat advanced colorectal carcinoma (18, 19); (4) cyclophosphamide, an alkylating agent that is employed against various neoplastic and autoimmune conditions (20–23); and (5) bortezomib, a proteasomal inhibitor approved for the therapy of multiple myeloma and mantle cell lymphoma (24–26). Specific forms of irradiation as well as photodynamic therapy, both of which are habitually employed for the treatment of various neoplasms, have also been shown to trigger *bona fide* ICD (27–34). Finally, a bunch of hitherto experimental agents is intrinsically endowed with the capacity to initiate ICD, including (but not limited to) some oncolytic viruses (35–39), the microtubular inhibitor patupilone (40–42), and elevated hydrostatic pressures (43).

According to accepted models, ICD relies on the establishment of adaptive stress responses that promote the spatiotemporally coordinated emission of endogenous danger signals from dying cells (44, 45). The endogenous molecules that dispatch danger signals in response to stress are cumulatively known as “damage-associated molecular patterns” (DAMPs) and operate upon binding to receptors expressed by bystander cells, including cellular components of both the innate and adaptive immune system (2, 46–49). As it stands, four DAMPs have been shown to be required for RCD as induced by anthracyclines to be perceived as immunogenic, namely, (1) the exposure of the endoplasmic reticulum (ER) chaperone calreticulin (CALR) on the outer surface of the plasma membrane (16); (2) the secretion of ATP (50); (3) the production of type I interferon (IFN) (51); and (4) the release of the non-histone chromatin-binding protein high-mobility group box 1 (HMGB1) into the extracellular space (52). This said, it cannot be formally excluded that other hitherto undiscovered DAMPs are required for anthracycline-elicited RCD to promote an adaptive immune response. Along similar lines, not all these DAMPs may be required for RCD as induced by agents other than anthracyclines to be perceived as immunogenic (53–55).

In this context, i.e., anthracycline-induced ICD, CALR exposure obligatorily relies on the establishment of a pre-mortem ER stress response centered around the phosphorylation of eukaryotic translation initiation factor 2A, 65 kDa (EIF2A) (7, 56), ATP secretion requires the induction of autophagy (57), and type I IFN production stems from toll-like receptor 3 (TLR3) signaling (51). The molecular mechanisms underlying the ability of anthracyclines and other ICD inducers to promote HMGB1 release remain obscure (2, 3). Cumulatively, these DAMPs recruit antigen-presenting cells (APCs) to sites of active ICD and stimulate the uptake, processing, and presentation of dead cell-associated antigens, eventually resulting in the priming of an adaptive immune response (2, 3). In particular, CALR promotes antigen uptake by APCs by binding to low density lipoprotein receptor-related protein 1 (LRP1, best known as CD91) (58); ATP stimulates the recruitment of APCs and their activation upon binding to purinergic receptor P2Y, G-protein coupled, 2 (P2RY2) and purinergic receptor P2X, ligand-gated ion channel, 7 (P2RX7), respectively (50, 59, 60); type I IFNs exert immunostimulatory effects via IFN (alpha, beta, and omega) receptors (IFNARs) (51); and HMGB1 does so through TLR4 and advanced glycosylation end product-specific receptor (AGER, best known as RAGE) (52, 61).

A detailed discussion of the molecular and cellular mechanisms involved in the detection of ICD-associated DAMPs goes beyond the scope of this review and can be found in Ref. (2, 3). However, it is important to note that the failure of cancer cells to emit one (or more) of these DAMPs completely compromises the immunogenicity of RCD (2, 3). Thus, at odds with their wild-type counterparts, *Calr^−/−^* murine CT26 colorectal cells exposed to anthracyclines are unable to vaccinate mice against a subsequent inoculation with malignant cells of the same type (16). The same holds true in several other situations in which adaptive responses cannot proceed normally, including the genetic inhibition of autophagy (e.g., upon the expression of short-hairpin RNAs targeting the essential autophagy proteins Atg5 or Atg7) or the unfolded protein response (e.g., upon the expression of a non-phosphorylatable variant of EIF2A) (7, 57, 62, 63).

Accumulating clinical evidence indicates that the (re-)activation of a proficient immune response against malignant cells is associated with improved disease outcome in patients affected by a wide panel of neoplasms (64–68), in particular when malignant lesions are highly infiltrated by immune effector cells prior to therapy (69). Considerable efforts are therefore being devoted to the development of clinically implementable strategies that (re-)instate anticancer immunosurveillance (70, 71). So far, the most successful of these approaches involves the administration of monoclonal antibodies (mAbs) that block immunosuppressive receptors expressed by activated T cells, such as cytotoxic T lymphocyte-associated protein 4 (CTLA4) and programed cell death 1 (PDCD1, best known as PD-1) (72, 73). Three distinct checkpoint blockers of this type, namely, the CTLA4-targeting mAb ipilimumab and the PD-1-targeting mAbs nivolumab and pembrolizumab, are approved by the US Food and Drug Administration and other regulatory agencies worldwide for use as standalone immunotherapeutic interventions in melanoma patients (74–77). In addition, the administration of checkpoint blockers has been shown to improve the clinical profile of various chemotherapeutic and immunotherapeutic agents (78). Along similar lines, various combinatorial immuno(chemo)therapeutic regimens are being investigated in clinical trials for their ability to mediate superior antineoplastic effects as compared to monotherapies based on their constituents (79, 80). In this framework, various attempts are being made to render immunogenic otherwise non-immunogenic instances of therapy-induced RCD, thereby converting them into *bona fide* ICD (79, 81–84). This can be due to molecular defects that prevent cancer cells from emitting DAMPs appropriately, as mentioned above, as well as to the intrinsic features of the therapeutic agent under consideration (Table 1). For instance, at odds with its derivative oxaliplatin, cisplatin is intrinsically unable to trigger ICD since it does not stimulate the exposure of CALR on the outer surface of the plasma membrane (18, 19, 85).

**Table 1:** **Immunogenicity of chemotherapy-induced regulated cell death (examples)**.

Drug	CALR exposure	ATP secretion	Type I IFN production	HMGB1 release	^a^*Bona fide* ICD inducer	Restoration of ICD	Reference
5-Fluorouracil	Debated	No	n.d.	Yes	n.d.	RT	(16)
							(86)
							(87)

Bleomycin	Yes	Yes	Yes	Yes	Yes	n.a.	(88)

Bortezomib	Yes	n.d.	Yes	Yes	Yes	n.a.	(24)
							(25)
							(26)
							(89)

Camptothecin	Debated	No	n.d.	Yes	No	n.d.	(16)
							(87)

Carboplatin	Partial	Yes	n.d.	Partial	No	RT	(16)
							(86)

Cisplatin	No	Yes	n.d.	Yes	No	Pyridoxine	(19)
						Thapsigargin	(90)
						Tunicamycin	(91)
						ZnCl_2_	(92)
							(93)
							(94)

Cyclophosphamide	Yes	Yes	Yes	Yes	Yes	n.a.	(20)
							(21)
							(95)

Digitoxin	Yes	Yes	n.d.	Partial	No	Cytotoxic agents	(81)
Digoxin							(83)

Docetaxel	Yes	No	n.d.	No	No	n.d.	(96)
							(97)

Doxorubicin	Yes	Yes	Yes	Yes	Yes	n.a.	(15)
							(16)
							(17)
							(51)
							(98)
							(99)

Epirubicin	Yes	Yes	n.d.	Yes	Yes	n.a.	(16)
							(17)

Etoposide	No	Yes	n.d.	Yes	No	Calyculin A	(16)
						Salubrinal	(17)
						Tautomycin	(93)
						PP1/GADD34-targeting peptides	(100)
						2-deoxyglucose	(101)
							(102)

Gemcitabine	No	Partial	n.d.	Yes	No	PX-478	(103)

Idarubicin	Yes	n.d.	n.d.	Yes	Yes	n.a.	(17)
							(16)
							(104)

Irinotecan	n.d.	n.d.	n.d.	Yes	n.d.	n.d.	(105)

Mafosfamide	Yes	n.d.	n.d.	Yes	Yes	n.d.	(20)
Melphalan	Debated	n.d.	n.d.	Yes	n.d.	n.d.	(106)
							(107)
							(108)

Mitomycin C	Debated	No	n.d.	Yes	No	n.d.	(16)
							(87)

Mitoxantrone	Yes	Yes	Yes	Yes	Yes	n.a.	(7)
							(16)
							(17)
							(51)
							(57)
							(93)
							(109)

Oxaliplatin	Yes	Yes	Yes	n.d.	Yes	n.a.	(7)
							(18)
							(52)
							(57)
							(93)
							(110)

Patupilone	Yes^b^	n.d.	Yes	Yes^b^	Yes^b^	n.a.	(41)
							(42)

Temozolomide	No	Yes	n.d.	Yes	n.d.	Oncolytic virotherapy	(111)
						Cyclophosphamide	(112)

Vemurafenib	Yes	n.d.	n.d.	Yes	n.d.	n.d.	(103)
							(113)

*CALR, calreticulin; HMGB1, high-mobility group box 1; ICD, immunogenic cell death; IFN, interferon; n.a., not applicable; n.d., not determined; RT, radiation therapy*.

*^a^As determined in gold-standard vaccination experiments*.

*^b^Unpublished observations from our group*.

Here, we discuss strategies to convert non-immunogenic instances of RCD into *bona fide* ICD. In particular, we will review approaches for (1) correcting the incapacity of some therapeutic agents to kill cancer cells while provoking the emission of one or more DAMP(s); or (2) complementing the missing DAMP(s) with exogenous interventions. On the contrary, we will not dwell on strategies that boost the immunogenicity of RCD by operating downstream of DAMP-sensing receptors.

## Combinatorial Strategies to Restore CALR Exposure

Some anticancer therapeutics efficiently kill cancer cells (hence promoting the release of HMGB1) and stimulate the secretion of both ATP and type I IFNs, but selectively fail to promote CALR exposure. Most often, such a defect originates from the inability of these agents to trigger an ER stress response resulting in EIF2A phosphorylation (56, 114), and hence can be corrected by the co-administration of an ER stressors. As mentioned above, cisplatin is one of the antineoplastic agents that fail to trigger *bona fide* ICD as it does not drive a robust ER stress response (18, 19, 85). The ER-stressing agents that have been shown to correct this defect, hence rendering cisplatin-induced RCD immunogenic, include thapsigargin, an inhibitor of various members of the sarco/endoplasmic reticulum Ca^2+^-ATPase (SERCA) (19, 114); tunicamycin, an inhibitor of *N*-glycosylation (19, 94, 114); pyridoxine, a cell-permeant precursor of bioactive vitamin B6 (90, 91, 115); and ZnCl_2_ (92). Similar results have been obtained by establishing an ER stress response through the enforced overexpression of reticulon 1 (RTN1), an ER protein involved in vesicular trafficking and secretion (116, 117). The latter approach is obviously incompatible with clinical applications. Nonetheless, these data reinforce the notion that the immunogenicity of cisplatin-induced RCD can be restored by various interventions that induce an ER stress (94).

Another strategy that successfully restores CALR exposure in cells succumbing to chemicals that *per se* do not enable this phenomenon consists in the co-administration of inhibitors of the EIF2A phosphatase composed of protein phosphatase 1, regulatory subunit 15A (PPP1R15A, best known as GADD34), and pyrophosphatase (inorganic) 1 (PPA1, best known as PP1), resulting in accrued EIF2A phosphorylation even in the absence of overt ER stress (16). Thus, whereas CT26 cells treated with etoposide (a topoisomerase II inhibitor currently approved for the treatment of various malignancies) (118, 119) do not expose CALR as they die, and hence fail to vaccinate mice against a subsequent challenge with neoplastic cells of the same type, they efficiently do so in the presence of tautomycin, calyculin A, and salubrinal (three distinct GADD34/PP1 inhibitors) (16). Similar results have been obtained with the small-interfering RNA (siRNA)-mediated downregulation of PP1 or GADD34 (16), as well as with short cell-permeant peptides that disrupt the physical interaction between these two proteins (102). Although siRNA- and peptide-based strategies may not be easily implemented in clinical settings, these results corroborate the specificity of tautomycin, calyculin A, and salubrinal, and lend further support to the notion that interventions that stimulate EIF2A phosphorylation efficiently promote CALR exposure even in the absence of overt ER stress (120).

At least theoretically, the co-administration of ER stressors or molecules that promote EIF2A phosphorylation can be harnessed to reconstitute the immunogenicity of RCD induced by all anticancer agents that *per se* do not stimulate CALR exposure on the cell surface but provoke ATP secretion, type I IFN production, and HMGB1 release. In addition, the inability of some anticancer agents to cause the translocation of CALR to the outer leaflet of the plasma membrane can be corrected, at least in some settings, by the co-administration of exogenous, recombinant CALR (7, 16, 106). CALR is indeed relatively “sticky” and its absorption on malignant cells succumbing to non-immunogenic RCD *in vitro* has been shown to fully restore the ability of these cells to vaccinate syngeneic mice against a subsequent neoplastic challenge (16). To the best of our knowledge, however, whether the systemic or intratumoral administration of recombinant CALR to tumor-bearing mice treated with non-immunogenic therapeutics is able to convert them into *bona fide* ICD inducers has not been tested yet. As compared to administration of small molecules that establish an ER stress response or promote EIF2A phosphorylation, the use of recombinant CALR appears advantageous in that (at least theoretically) it would complement the lack of CALR exposure in all scenarios, irrespective of the underlying molecular defects (including the downregulation or loss of CALR itself). However, such an approach may not be implementable in the clinic, owing to pharmacodynamic and pharmacokinetic issues (e.g., distribution of the recombinant protein, serum half-life, etc…) as well as economic considerations. Current efforts are therefore being focused on the identification of novel (and the refinement of existing) small molecule-based strategies to stimulate CALR exposure upon the establishment of an ER stress or the induction of EIF2A phosphorylation.

## Combinatorial Strategies to Boost ATP Secretion

In some settings, anticancer agents kill malignant cells in an efficient fashion (which corresponds to a consistent release of HMGB1), while stimulating the exposure of CALR and the production of type I IFN, but this is not accompanied by the accumulation of extracellular ATP (57, 121), a defect that can stem from at least three different causes. First, some therapeutic agents are unable to stimulate (or even inhibit) autophagic responses, which are required for dying cells to secrete ATP in sufficient amount for signaling via P2RY2 and P2RX7 receptors (57). Second, some malignant cells bear genetic or epigenetic defects that affect the molecular machinery for autophagy (122, 123). These cells are intrinsically unable to preserve the intracellular ATP pool in the course of stress responses, resulting in limited ATP secretion during death (124). Third, some neoplastic cells express high levels of either ectonucleoside triphosphate diphosphohydrolase 1 (ENTPD1, best known as CD39) or 5′-nucleotidase, ecto (NT5E, best known as CD73), two membrane-bound nucleotidases that degrade extracellular ATP (125).

So far, one general strategy has been shown to restore extracellular ATP concentrations to levels that are compatible with the efficient recruitment and activation of APCs, namely, the pharmacological inhibition of CD39. Thus, CT26 cells lacking essential components of the autophagic machinery, such as Atg5, Atg7, or Beclin 1 (Becn1), secrete limited amounts of ATP as they succumb to anthracyclines, and hence are incapable of vaccinating syngeneic mice against a subsequent challenge with malignant cells of the same type (57). Such a functional defect can be corrected by the co-administration of ARL67156, a broad spectrum inhibitor of extracellular nucleotidases (57). Further confirming these findings, CT26 engineered to overexpress CD39 and exposed to anthracyclines are unable to protect syngeneic mice against a subsequent injection with neoplastic cells of the same type (57, 125). This defect can be corrected by the co-administration of ARL67156, along with the restoration of RCD-associated ATP secretion (57, 125). Taken together, these results indicate that inhibitors of extracellular nucleotidases may constitute a convenient manner to boost the immunogenicity of RCD instances that are normally not associated with ATP secretion.

Importantly, the pharmacological activation of autophagy does not suffice for cancer cells to become immunogenic (16, 57). Nonetheless, combining anticancer agents that *per se* are unable to trigger ATP secretion with molecules that upregulate the autophagic flux, such as inhibitors of mechanistic target of rapamycin (MTOR) complex I (MTORCI), may efficiently convert non-immunogenic RCD instances into *bona fide* ICD. This hypothesis awaits formal experimental confirmation. Indeed, while other inducers of autophagy such as the glycolytic inhibitor 2-deoxyglucose (126) have been shown to reinstate the immunogenicity of etoposide-elicited RCD, such an effect was ascribed to the restoration of CALR exposure (indeed, etoposide kills malignant cells while promoting ATP secretion) (100). Finally, it should be noted that the establishment of an ATP gradient around dying cells may not constitute a general requirement for the perception of RCD as immunogenic (127). Moreover, at least in some settings, autophagy may actually inhibit ICD by limiting the production of reactive oxygen species in the course of adaptive stress responses, hence counteracting the establishment of ER stress and consequent CALR exposure (54, 55). Thus, further work is required to precisely identify malignancies in which autophagy supports ICD. Only in these scenarios, the co-administration of autophagy inducers may constitute a proper approach to reinstate the immunogenicity of RCD.

## Combinatorial Strategies to Promote Type I IFN Production

Whereas the role of type I IFN in the regulation of innate and adaptive immune responses is well known (128, 129), type I IFN signaling in malignant cells has been identified as a requirement for (anthracycline-induced) ICD only recently (51). Thus, cancer cells respond to various anthracyclines by activating a TLR3-elicited signal transduction cascade resulting in type I IFN release, autocrine/paracrine type I IFN signaling, and chemokine (C–X–C motif) ligand 10 (CXCL10) secretion, two phenomena that underlie their vaccinating potential. At odds with their wild-type counterparts, *Tlr3^−/−^* and *Ifnar1^−/−^* murine cancer cells exposed to anthracyclines fail to vaccinate syngeneic mice against a subsequent injection of living cells of the same type (51). It has already been demonstrated that the inability of *Tlr3^−/−^* cells to undergo ICD can be corrected by the co-administration of recombinant type I IFNs or recombinant CXCL10. Similarly, *Ifnar1^−/−^* cells succumbing to anthracyclines turn immunogenic in the presence of recombinant CXCL10 (but not type I IFNs) (51).

Various synthetic TLR3 agonists are available and some of them, including polyinosinic:polycytidylic acid (polyI:C) and its clinical grade analog polyI:polyC12U (also known as rintatolimod and Ampligen™), have been extensively tested as immunostimulants in cancer patients (130, 131). It is therefore tempting to speculate that the co-administration of TLR3 agonists may restore the ability of anticancer agents that *per se* do not promote type I IFN release to trigger *bona fide* ICD. This hypothesis awaits urgent experimental confirmation. For the considerations presented above, small molecules that trigger TLR3 signaling would indeed be more convenient as clinical tools to restore type I IFN signaling than recombinant type I IFN or CXCL10 themselves.

## Combinatorial Strategies to Substitute for HMGB1 Release

HMGB1 release occurs upon (nuclear and) plasma membrane permeabilization, i.e., it constitutes a post-mortem event (5, 132). Thus, all antineoplastic agents that efficiently kill malignant cells (as opposed to molecules that exert cytostatic effects or induce cell senescence) (133) promote HMGB1 release, perhaps with different kinetics (5, 132). However, the expression levels of HMGB1 vary in different tumor types and evolve along with tumor progression, implying that some malignant cells may express HMGB1 to levels that are not compatible with the activation of TLR4 and RAGE in immune cells upon release (134, 135). Importantly, the immunogenicity of anthracycline-induced RCD is compromised in these cells, as well in cells artificially depleted of HMGB1 by means of specific siRNAs (135). Recent results indicate that this defect can be efficiently corrected by the exogenous supply of a synthetic TLR4 agonist, i.e., dendrophilin, at least in experimental models (135). Since dendrophilin has not yet entered clinical development (130, 131), it will be interesting to see whether TLR4 agonists that are already licensed by regulatory agencies for use in humans, such as the Bacillus Calmette–Guérin (BCG) (80) and monophosphoryl lipid A (MPL) (136), are also able to restore the immunogenicity of HMGB1-deficient cells succumbing to ICD.

In this context, it is worth noting that cancer cells exposing CALR, secreting ATP, producing type I IFNs but releasing limited amounts of HMGB1 as they respond to a lethal stimulus in a suboptimal manner fail to elicit adaptive immune responses (137). Upon inoculation into immunocompetent mice, these cells actually form tumors at the vaccination site (as a significant fraction of them is not dying) and the animals are unable to control a subsequent challenge with cell of the same type (3). We have observed this to occur in murine cancer cells treated with digoxin or digitoxin, two glycosides approved in many countries for the treatment of cardiac conditions (81). These molecules efficiently inhibit the human Na^+^/K^+^ ATPase, which explains their pharmacological properties and their ability to kill some neoplastic cells of human origin, but not its murine counterpart (83). Thus, cardiac glycosides *per se* are unable to trigger ICD, at least in the murine system. However, clinical data indicate that they may convert non-immunogenic RCD as elicited by a very large panel of chemotherapeutics into *bona fide* ICD (83). From another standpoint, any anticancer agent that efficiently kills malignant cells could be considered as a means to restore the immunogenicity of cells responding to cardiac glycosides. We have recently initiated a clinical trial to prospectively test this hypothesis in head and neck squamous carcinoma patients.

## Concluding Remarks

**Figure 1:**
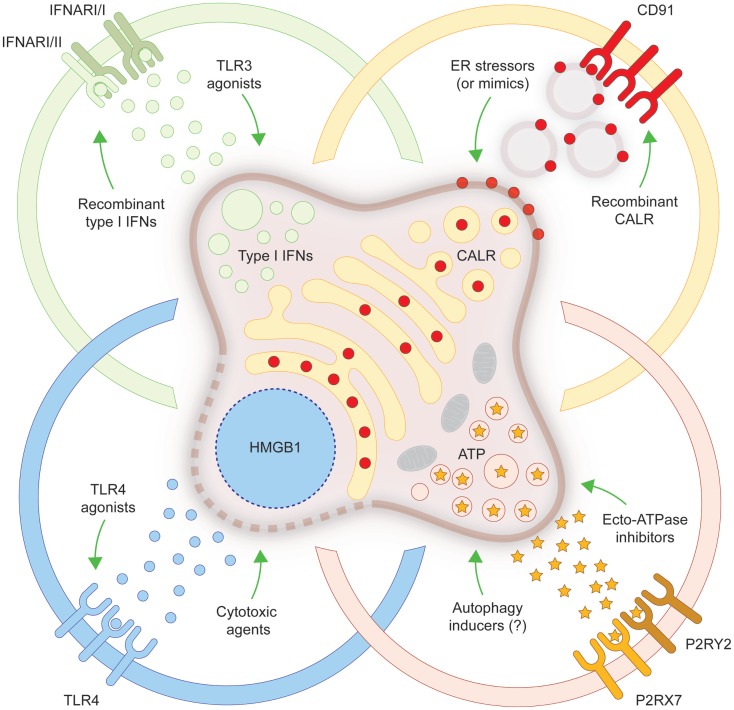
**Strategies to convert non-immunogenic RCD into *bona fide* ICD**. Upon inoculation into immunocompetent syngeneic hosts, cancer cells responding to a panel of lethal stimuli trigger an adaptive immune response against dead cell-associated antigens. Such an immunogenic variant of regulated cell death (RCD), commonly known as immunogenic cell death (ICD), relies on the exposure of calreticulin (CALR) on the cell surface, on the secretion of ATP, on the production of type I interferons (IFNs) and on the release of high-mobility group box 1 (HMGB1, which accompanies cell death). When any of these damage-associated molecular patterns cannot be emitted (in the appropriate spatiotemporal order), dying cancer cells cannot be perceived anymore as immunogenic by the host immune system. Several strategies have been conceived to correct these defects, hence converting non-immunogenic RCD into *bona fide* ICD. ER, endoplasmic reticulum; IFNAR, interferon (alpha, beta, and omega) receptor; P2RX7, purinergic receptor P2X, ligand gated ion channel, 7; P2RY2, purinergic receptor P2Y, G-protein coupled, 2; TLR, toll-like receptor.

In spite of old beliefs, cancer cells continuously interact with the immune system: first, as they are generated by healthy cells upon malignant transformation; second, as they evolve and acquire additional neoplastic features; and third, when they are challenged with therapeutic interventions. During the last decade, such a conceptual revolution, i.e., considering tumors as entities that can be detected and destroyed by the immune system, has paved the way toward the development of novel therapeutic agents conceived to re(instate) anticancer immunity, and some of these interventions have already been licensed for use in humans by international regulatory agencies. In addition, it has become clear that many therapeutics that had been used for decades in the clinic are efficient (for the most part) because they engage the host immune system against malignant cells. ICD is one of the several mechanisms through which cytotoxic chemotherapeutics, targeted anticancer agents as well as some forms of radiotherapy can elicit tumor-targeting immune responses. Identifying novel ICD inducers as well as measures that convert non-immunogenic RCD into *bona fide* ICD is of primordial importance. Promising preclinical results and preliminary clinical findings suggest, indeed, that agents that promote CALR exposure, ATP secretion, type I IFN production, HMGB1 release or stimulate the downstream signal transduction pathway may considerably improve the clinical profile of conventional therapeutic regimens (Figure 1). A systematic investigation of the ability of currently available anticancer agents to elicit the abovementioned ICD-associated processes in human cancer cells of distinct histological origin is urgently awaited. These data may pave the way to the clinical implementation of combinatorial immuno(chemo)regimens that efficiently promote ICD and hence mediate complete tumor regression in a high proportion of patients.

## Conflict of Interest Statement

The authors declare that the research was conducted in the absence of any commercial or financial relationships that could be construed as a potential conflict of interest. The Guest Associate Editor, Patrizia Agostinis, declares that despite having co-authored a few manuscripts with the authors Lorenzo Galluzzi, Oliver Kepp and Guido Kroemer in the past 2 years, there has been no conflict of interest during the review and handling of this manuscript.

## Abbreviations

APC, antigen-presenting cell; CALR, calreticulin; CTLA4, cytotoxic T lymphocyte-associated protein 4; CXCL10, chemokine (C–X–C motif) ligand 10; DAMP, damage-associated molecular pattern; EIF2A, eukaryotic translation initiation factor 2A, 65 kDa; ER, endoplasmic reticulum; HMGB1, high-mobility group box 1; ICD, immunogenic cell death; IFN, interferon; IFNAR, interferon (alpha, beta, and omega) receptor; mAb, monoclonal antibody; P2RX7, purinergic receptor P2X, ligand gated ion channel, 7; P2RY2, purinergic receptor P2Y, G-protein coupled, 2; RCD, regulated cell death; siRNA, small-interfering RNA; TLR, toll-like receptor.
