# Corrigendum: “Combinatorial Strategies for the Induction of Immunogenic Cell Death”

**DOI:** 10.3389/fimmu.2015.00275

**Published:** 2015-06-01

**Authors:** Lucillia Bezu, Ligia C. Gomes-da-Silva, Heleen Dewitte, Karine Breckpot, Jitka Fucikova, Radek Spisek, Lorenzo Galluzzi, Oliver Kepp, Guido Kroemer

**Affiliations:** ^1^Equipe 11 Labellisée par la Ligue Nationale Contre le Cancer, Centre de Recherche des Cordeliers, Paris, France; ^2^U1138, INSERM, Paris, France; ^3^Metabolomics and Cell Biology Platforms, Gustave Roussy Campus Cancer, Villejuif, France; ^4^Faculté de Medecine, Université Paris-Sud, Le Kremlin-Bicêtre, France; ^5^Department of Chemistry, University of Coimbra, Coimbra, Portugal; ^6^Laboratory for General Biochemistry and Physical Pharmacy, Faculty of Pharmacy, Ghent University, Ghent, Belgium; ^7^Laboratory of Molecular and Cellular Therapy, Vrije Universiteit Brussel, Jette, Belgium; ^8^Sotio a.c., Prague, Czech Republic; ^9^Department of Immunology, 2nd Faculty of Medicine, University Hospital Motol, Charles University, Prague, Czech Republic; ^10^Gustave Roussy Campus Cancer, Villejuif, France; ^11^Université Paris Descartes, Paris, France; ^12^Université Pierre et Marie Curie, Paris, France; ^13^Pôle de Biologie, Hopitâl Européen George Pompidou, AP-HP, Paris, France

**Keywords:** ATP, autophagy, calreticulin, endoplasmic reticulum stress, HMGB1 protein, type I interferon

We realized that the family name of one of the authors was misspelled in the original version of the article. Indeed, the name of the co-first author is not Ligia C. Gomes-de-Silva, but Ligia C. Gomes-da-Silva. We apologize to this author and to the readers of *Frontiers in Immunology* for the inconvenience this may have caused.

The original article has been updated.

## Conflict of Interest Statement

The authors declare that the research was conducted in the absence of any commercial or financial relationships that could be construed as a potential conflict of interest.

